# The Role of the Insulin/IGF System in Cancer: Lessons Learned from Clinical Trials and the Energy Balance-Cancer Link

**DOI:** 10.3389/fendo.2015.00077

**Published:** 2015-05-15

**Authors:** Laura W. Bowers, Emily L. Rossi, Ciara H. O’Flanagan, Linda A. deGraffenried, Stephen D. Hursting

**Affiliations:** ^1^Department of Nutrition, University of North Carolina at Chapel Hill, Chapel Hill, NC, USA; ^2^Department of Nutritional Sciences, University of Texas at Austin, Austin, TX, USA

**Keywords:** insulin-like growth factor, insulin, biomarkers, obesity, energy balance

## Abstract

Numerous epidemiological and pre-clinical studies have demonstrated that the insulin/insulin-like growth factor (IGF) system plays a key role in the development and progression of several types of cancer. Insulin/IGF signaling, in cooperation with chronic low-grade inflammation, is also an important contributor to the cancer-promoting effects of obesity. However, clinical trials for drugs targeting different components of this system have produced largely disappointing results, possibly due to the lack of predictive biomarker use and problems with the design of combination therapy regimens. With careful attention to the identification of likely patient responders and optimal drug combinations, the outcome of future trials may be improved. Given that insulin/IGF signaling is known to contribute to obesity-associated cancer, further investigation regarding the efficacy of drugs targeting this system and its downstream effectors in the obese patient population is warranted.

## Introduction

The insulin and insulin-like growth factor (IGF) signaling system, a key regulator of growth and energy metabolism, is involved in the pathogenesis and progression of numerous malignancies. Population studies have clearly established a link between insulin and IGF-I levels and the risk and prognosis of certain cancers, with much of this research driven by the rapidly growing incidence of obesity and metabolic syndrome across the globe (1–5). While a number of factors likely contribute to the increased cancer risk and cancer-related mortality that accompanies obesity, dysregulated insulin and IGF signaling is thought to play a significant role. Epidemiological evidence linking the insulin/IGF system with cancer has been reinforced by a large body of pre-clinical work in cell culture and animal models that has established many of the mechanisms underlying these associations.

Consequently, enthusiasm regarding the development and testing of pharmaceutical agents targeting the type 1 IGF receptor (IGF-IR) was very high in the 1990s and early 2000s. However, while the early phase clinical trials for these agents showed some promise, large randomized phase III trials failed to demonstrate that the addition of these drugs to a conventional treatment regimen results in a significant clinical benefit (6, 7), and many drug development programs targeting IGF-IR were shut down. Several researchers have argued that these programs were abandoned too quickly, though. They suggest that the poor phase III trial results were due to a number of factors, including the failure to use predictive biomarkers for the identification of probable responders and non-optimal drug combinations and timing (8, 9). With greater attention to these considerations and a better understanding of the complex insulin/IGF system, future trials may have more success. Drugs targeting other components of this system (alone or in combination), including the insulin receptor (IR) as well as various ligands and downstream signaling molecules, may also prove to be more efficacious.

This review will examine how dysregulation of the insulin/IGF system, which occurs with obesity and metabolic syndrome, promotes cancer risk and progression. Several cancer prevention and treatment interventions targeting insulin/IGF signaling or downstream factors will also be described, as well as recent results from trials testing these agents. Finally, following an analysis of the lessons learned from previous clinical trials, we will conclude with a discussion of potential new strategies, including both pharmaceutical and lifestyle interventions. However, in order to understand the links between dysregulated insulin/IGF signaling and cancer as well as the variety of ideas regarding how to most effectively block this signaling, one must first appreciate the system’s complexity. We will thus begin with an overview of the structure and functions of the components of the insulin/IGF system.

## The Insulin/IGF Signaling System

### Receptors

The IR and IGF-IR are transmembrane tyrosine kinase receptors with a high degree of homology (10). Their functions also partially overlap and can vary depending on cell type. However, the IR is traditionally considered a regulator of metabolism, specifically the storage and release of glucose, protein, and lipids, while the IGF-IR controls whole body and organ growth. Both are heterotetramers composed of two half receptors formed when the receptor gene products are processed to glycosylated alpha and beta subunits that associate together. The extracellular alpha subunit contains the ligand-binding domain while the transmembrane beta subunit possesses the tyrosine kinase domain (11). The exception to this structural homology among the receptors is the type 2 IGF receptor (IGF-IIR), which lacks tyrosine kinase function. This receptor instead serves to clear IGF-II from circulation by binding and internalizing its ligand, which is then subject to lysosomal degradation (12). There are also two forms of the IR that are generated by alternate splicing of exon 11 in the receptor’s gene. IR-A results from the exclusion of exon 11 and has greater mitogenic function than IR-B, which is formed when exon 11 is included and has greater metabolic function. Both IR-A and IGF-IR are ubiquitously expressed in normal adult tissues, but IR-B expression is typically limited to metabolic tissues like the liver, muscle, and adipose (13, 14). Hybrid receptors consisting of a half IR and a half IGF-IR can also form. Given that most cancer cells express both IR and IGF-IR, multiple homo- and heterodimer variations of these receptors may be found in tumors (11). However, gene amplification or mutations resulting in overexpression or ligand-independent activation of the insulin/IGF system receptors are rare (8).

### Ligands and binding proteins

**Figure 1:**
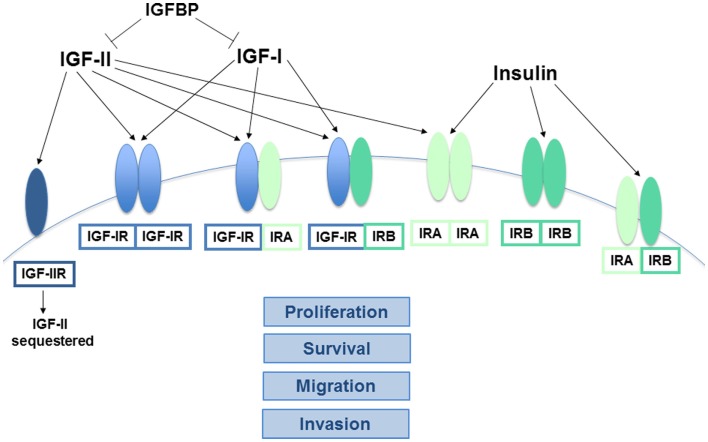
**Interactions between the key components of the insulin/IGF system**. The receptors of the insulin/IGF system are tetramers comprised of two half receptors, each with an extracellular ligand-binding domain and an intracellular tyrosine kinase domain. IGF-IIR is the exception, as it lacks a kinase domain. Both IGF-IR and IR homodimers and IGF-IR/IR heterodimers can form, depending on the relative abundance of the half receptors. Alternative splicing also results in two different forms of the IR half receptor, IR-A and IR-B. The ligands of this system (insulin, IGF-I, and IGF-II) vary in their affinity for the different receptors. IGF-I primarily binds to homo or heterodimers containing an IGF-IR half receptor, while insulin has the greatest affinity for IR-A and IR-B. In contrast, IGF-II binds IR-A with high affinity and can also bind IGF-IR homo or heterodimers, but binding to IGF-IIR limits its bioavailability. Similarly, the IGFBPs sequester both the IGFs, preventing their ability to bind and activate their cognate receptors. The signaling pathways downstream of the activated IR-A and IGF-IR homodimers are known to stimulate cancer cell proliferation, survival, migration, and invasion, while IR-B is more closely linked to metabolic regulation. The exact functions of the various heteroreceptor combinations have not been clearly defined, but it is likely that receptors containing an IR-A or IGF-IR holoreceptor will modulate cancer growth and metastasis to some degree.

**Figure 2:**
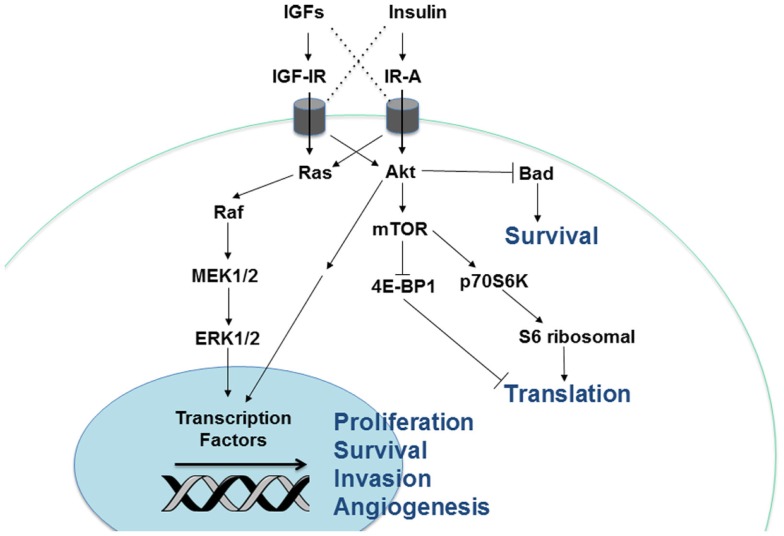
**Cellular signaling pathways downstream of the insulin/IGF receptors**. Insulin and IGF activate two major signaling pathways, Akt and Ras-MAPK. Stimulation of Akt activates the mTOR signaling complex, leading to greater protein translation. In addition, Akt and Ras-MAPK enhance cellular proliferation, survival, angiogenesis, and invasion via regulation of gene transcription.

In addition to the four receptors described above, the insulin/IGF system involves three ligands: insulin, IGF-I, and IGF-II. Insulin is produced by pancreatic beta cells and primarily in response to elevated blood glucose levels. IGF expression is more widespread, but the liver is the predominant site of production. IGF-I expression in the liver is stimulated by growth hormone (GH), but tissue-specific factors also play a role in its regulation elsewhere (8). Both genetic and lifestyle factors impact an individual’s circulating IGF-I levels to an approximately equal degree (15). IGF-II expression is also regulated by hormones and affected by lifestyle factors like obesity (16). Cancer cells can produce the IGFs, so while insulin must travel through circulation to reach a tumor, IGFs have the potential to interact with a cancer cell via autocrine, paracrine, and endocrine mechanisms (17). The insulin/IGF system receptors have varying affinities for these different ligands. While the metabolic IR-B primarily binds insulin only, IR-A binds insulin and IGF-II with equal affinity (18). IGF-IR preferentially binds the IGFs over insulin, but can bind all three. In addition, the IGFs can bind the IGF-IR/IR heterodimers (Figure 1). Ligand binding stimulates the kinase activity of these receptors via transphosphorylation of their beta subunits, resulting in phosphorylation of adaptor proteins, including the IR substrates (IRS 1–6) and Shc. These activate the phosphatidylinositol 3′-kinase (PI3K) and mitogen-activated protein kinase (MAPK) pathways, which regulate cell proliferation, survival, migration, metabolism, and angiogenesis (11) (Figure 2). Aberrant expression of the IGFs, particularly IGF-II (19), is common in many malignancies and may represent one mechanism by which the tumor stimulates its own growth. In fact, the primary purpose of the IGF-IIR, which lacks a tyrosine kinase domain, may be to counteract excessive IR-A and IGF-IR bioactivity by sequestering IGF-II.

Insulin-like growth factor bioactivity also has an additional level of regulation. Six IGF binding proteins (IGFBP) bind both IGF-I and IGF-II with high affinity and protect them from proteases while in circulation, which also effectively prevents their ability to interact with receptors until released (Figure 1). The IGFBPs must deliver the IGFs to a target tissue and dissociate from them via an enzymatic process in order to free them for activity. This suggests that the IGFBPs may act as tumor suppressors by limiting IGF activity, but conflicting study results and the fact that the IGFBPs are also thought to have IGF-independent effects on cancer (20) indicates that their role may be more complicated than originally hypothesized.

## Insulin/IGF Connection to Cancer

### Epidemiological evidence

Numerous studies have linked the insulin/IGF system with an increased risk of several cancers as well as a worse cancer prognosis, driving researchers’ interest in the enhancement of our understanding of these signaling pathways and the development of agents targeting them. For example, two prospective studies of postmenopausal women have found that hyperinsulinemia is associated with an elevated risk of breast cancer, though the link was limited to women not receiving hormone replacement therapy in one of the studies (21, 22). A meta-analysis of earlier population studies examining insulin levels and cancer incidence confirmed that hyperinsulinemia and high C-peptide levels (another measure of insulin production) are correlated with an increased risk of breast cancer as well as colorectal and pancreatic cancer (4). The link between serum IGF-I levels and cancer risk has also been investigated extensively. Intriguingly, a nested case-control study conducted within the Nurse’s Health Study found that an elevation in circulating IGF-I is associated with greater breast cancer risk in premenopausal women (23). Subsequent meta-analyses of several studies have substantiated these results (5, 24). Prostate cancer incidence has also been positively correlated with IGF-I in multiple prospective studies, with these findings confirmed by meta-analysis (5, 25, 26). These links appear to be independent of obesity-associated elevations in IGF-I, as obesity does not enhance prostate cancer or premenopausal breast cancer risk. Finally, researchers have demonstrated that both higher IGF-I and lower IGFBP-3 levels are associated with increased colorectal cancer risk in men and women (27, 28). Reports regarding IGFBP-3 levels and breast or prostate cancer risk have been contradictory, with some finding no association and others a positive or negative correlation (5, 23–26). These inconsistencies may be due to methodological issues and the complicated role that the IGFBPs seem to play in tumor growth, as argued by Renehan et al. (29). Regardless of the explanation, the highly variable results indicate that IGFBP levels are likely not an ideal indicator of cancer risk.

Researchers have also examined whether insulin/IGF signaling impacts cancer prognosis and have generally found that circulating insulin levels appear to be more predictive than the IGFs. This may be due to the relative importance of local IGF production and autocrine/paracrine signaling within the tumor. That is, systemic levels of the IGFs may not be as relevant to the growth of an established tumor that is capable of producing its own IGF-I and 2. In contrast, insulin is produced solely by the pancreatic beta cells, except in very rare cases, so serum insulin is a relatively good measure of the level of insulin signaling occurring at the tumor site. In fact, several prospective studies have demonstrated strong positive associations between different measures of insulin signaling and a poor breast cancer prognosis, including an increased risk of distant recurrence and mortality (1, 30, 31). Other investigators have shown similar correlations between high C-peptide levels and increased prostate and colorectal cancer mortality (2, 3). Breast tumor total IR levels have also been analyzed and found to be positively associated with poor survival (32, 33). In contrast, reports regarding the prognostic significance of IGF-IR levels in breast tumors are mixed, with some suggesting that its link to a better or worse outcome may depend on the breast cancer subtype (34–36). The reason(s) for these differences remain unclear. Greater tumor IGF-IR expression has been positively associated with a worse disease outcome for several other forms of cancer, including prostate cancer, gastric cancer, and renal cell carcinoma (37–39).

### Pre-clinical evidence

This epidemiological research has been accompanied by extensive mechanistic investigations that have established the pro-tumorigenic effects of insulin/IGF signaling. The mitogenic effect of insulin on mammary tumors was first demonstrated more than 40 years ago, when researchers found that insulin-deficiency reduced chemically induced mammary tumor growth in rats (40). Administration of exogenous insulin was also shown to reverse these effects (41). Researchers later determined that insulin has a similar effect on the growth of other forms of cancer (42, 43). These early findings may not have been pursued further because it was assumed that insulin signaling could not be targeted without the stimulation of unacceptable metabolic side effects. However, there has been renewed interest recently in the role of insulin in carcinogenesis, possibly due to the realization that resistance to IGF-IR inhibitors may be driven by continued IR signaling. In addition, small-molecule IR/IGF-IR inhibitors have been better tolerated metabolically than anticipated, suggesting that it is possible to target IR signaling without triggering hyperglycemia (8). In a recent series of papers, LeRoith and colleagues utilized a mouse model of genetically induced insulin resistance that develops hyperinsulinemia without obesity to examine how insulin signaling impacts mammary tumor growth *in vivo*. Using multiple models of mammary carcinoma, these investigators demonstrated that hyperinsulinemia promotes mammary tumor growth and pulmonary metastasis and that treatment with an insulin sensitizer or a small-molecule IR/IGF-IR inhibitor can attenuate these effects (44–47). Zhang et al. (48) have also shown that silencing the IR using shRNA blocks breast cancer cells’ ability to form pulmonary metastases *in vivo*. These results clearly illustrate the important role that insulin signaling can play in cancer growth and invasion.

Early experiments also validated the hypothesis that IGF-I signaling promotes tumorigenesis as well as cancer growth and invasion. In fact, IGF-IR expression was found to be necessary for the transforming action of the simian virus 40 large tumor antigen as well as numerous other oncogenes (49, 50). With these findings and indications that IGF-I stimulates cancer cell proliferation and metastasis (51–54), interest in this pathway as a potential pharmaceutical target grew. Animal models employing mutations that reduce circulating IGF-I have also confirmed that this growth factor does play a significant role in both mammary and prostate tumor growth *in vivo* (55, 56). IGF-IR monoclonal antibodies were the first IGF-I signaling drugs to be developed, and the pre-clinical studies testing these produced promising results that further reinforced earlier conclusions regarding this pathway’s important impact on tumorigenesis. An early study from Arteaga et al. (57) demonstrated that an IGF-IR monoclonal antibody inhibits the growth of human triple negative breast cancer cell xenografts in athymic mice. Other monoclonal antibodies targeting this receptor were later shown to inhibit the growth of multiple forms of cancer *in vivo*, including breast, pancreatic, renal, lung, and colon (58, 59). IGF-IR and dual IR/IGF-IR tyrosine kinase inhibitors (TKIs) have also been found to significantly attenuate the growth of several cancer cell lines (60–62). Overall, the pre-clinical evidence strongly supports the hypothesis that insulin/IGF signaling promotes the progression of numerous cancer types. The continued rise in global rates of obesity, which is typically accompanied by elevations in systemic insulin and IGF-I levels, suggests that efforts to develop pharmaceutical agents that reduce this signaling for cancer prevention and treatment are particularly warranted. The role that elevated or dysregulated insulin/IGF signaling, like that seen with obesity and other conditions, may play in the development of cancer will be discussed further below.

### Clinical trial evidence

**Figure 3:**
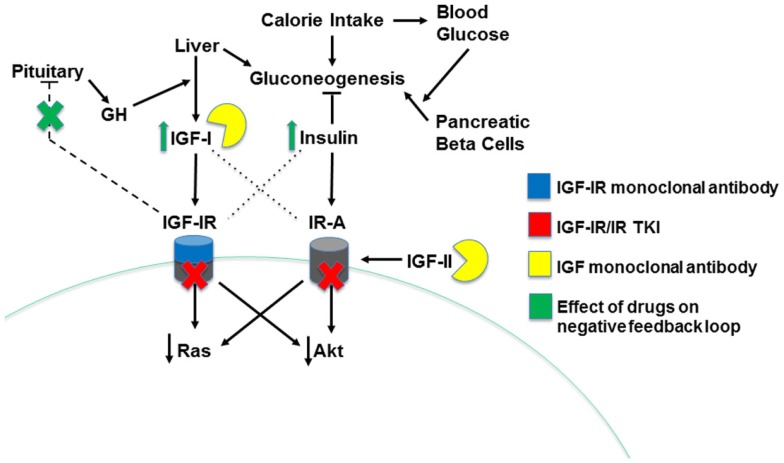
**Mechanisms of insulin/IGF system-targeting drugs**. The three types of insulin/IGF system-targeting drugs are illustrated. IGF-IR monoclonal antibodies bind IGF-IR, leading to its internalization and degradation. IGF-IR/IR TKI drugs decrease receptor activity by competing for the ATP binding site on the receptor’s kinase domain, blocking transduction of a signal to downstream effectors. Finally, IGF monoclonal antibodies directly bind both IGF-I and IGF-II, preventing them from binding and activating the system’s receptors. Drug-induced decreases in IGF-IR signaling disrupt the pituitary-mediated negative feedback loop regulating IGF-I production, leading to higher IGF-I levels and, indirectly, greater insulin levels.

Numerous pharmaceutical agents designed to specifically target the insulin/IGF system have been developed and tested for their tumor-inhibiting effects (Table 1). These can be divided into two general categories: receptor-targeting agents and drugs that reduce ligand bioactivity. The first includes both anti-receptor antibodies and small-molecule TKIs (Figure 3). Many of the agents that performed well in pre-clinical studies and early clinical trials have been further tested in phase II and III trials, with largely disappointing results that led to the discontinuation of many insulin/IGF-targeting programs. However, several experts in the field of insulin/IGF signaling have suggested that these findings do not indicate that this system is a poor cancer treatment target. They argue that these studies suffered from flaws in design, including a failure to use predictive biomarkers and to identify optimal drug combinations based on a clear understanding of insulin/IGF signaling (8, 9). Here, we will review the most current clinical trial results and recent developments based on the lessons learned from these.

**Table 1:** **Insulin/IGF-targeting drug trials**.

Agent name	Sponsor	Cancer types	Testing stage^a^	Reference^b^
**IGF-IR monoclonal antibodies**				
AVE1642	Sanofi	Advanced solid tumors	Discontinued	(69, 70)
Cixutumumab	NCI	Hepatocellular	Phase I/II	(83)
		Pancreatic		(84)
		Sarcomas		(85, 86)
		Thymic epithelial		(87)
Dalotuzumab	Merck	Advanced solid tumors	Phase I/II	(88, 89)
		Neuroendocrine		(91)
		NSCLC		(90)
Figitumumab	Pfizer	NSCLC	Discontinued	(6, 7, 65)
		Prostate		(64)
Ganitumab	Amgen	Advanced solid tumors	Phase I/II	(76)
		Breast		(79)
		Colorectal		(77, 78)
		Ewing family/desmoplastic		(82)
		Small round cell		
		Neuroendocrine		(80)
		Pancreatic		(81)
R1507	Hoffmann	NSCLC	Discontinued	(73)
	La Roche	Sarcomas		(72)
Robatumumab	Merck	Colorectal	Discontinued	(71)
**IGF-IR/IR TKIs**				
AXL 1717	Axelar	Astrocytomas	Phase I/II	
		Lung adenocarcinoma		
		NSCLC		(104)
		Squamous cell carcinoma		
BMS-754807	Bristol-Myers	Advanced solid tumors	Phase I/II	
	Squibb
		Breast		
OSI-906	Astellas	Advanced solid tumors	Phase I/II	(95, 96)
		Colorectal		(97)
**IGF monoclonal antibodies**				
BI 836845	Boehringer	Advanced solid tumors	Phase I/II	
	Ingelheim	Breast		
		NSCLC		
		Prostate		
MEDI-573	MedImmune	Advanced solid tumors	Phase I/II	(108, 109)
		Breast		
		Hepatocellular		

*^a^Testing stage indicates phase of current trials or most recent trials that have been conducted*.

*^b^Absence of a reference number indicates that the trial(s) for the indicated drug and cancer type are ongoing or have been completed but not published. Only published trials are reported for the IGF-IR monoclonal antibodies and OSI-906 due to the large number of trials for these drugs. IGF, insulin-like growth factor; IGF-IR, insulin-like growth factor I receptor; IR, insulin receptor; NSCLC, non-small cell lung cancer; TKI, tyrosine kinase inhibitor*.

#### Receptor-Targeting Agents: IGF-IR Monoclonal Antibodies

These agents prevent ligand activation of the IGF-IR by binding the receptor, which leads to its internalization and degradation. While they do not cross-react with the IR, they can bind and inhibit hybrid receptor activity (63). Figitumumab, an IGF-IR monoclonal antibody produced by Pfizer, is perhaps the most well-known member of this drug class. Despite promising phase II trial data (64, 65), phase III trials examining the combination of figitumumab with cytotoxic chemotherapy or the epidermal growth factor receptor (EGFR) inhibitor erlotinib for non-small cell lung cancer (NSCLC) were terminated early due to the drug’s failure to improve overall survival (OS) and problems with toxicity (6, 7). In addition, some phase II results were later retracted (66), and Pfizer discontinued its development of figitumumab. Unfortunately, several other IGF-IR antibodies have met a similar fate. AVE1642 (Sanofi) inhibited mammary tumor growth and metastases in pre-clinical studies (67, 68) and exhibited anti-tumor activity in phase I trials for patients with advanced solid tumors (69, 70). However, it failed in phase II trials, and its development was discontinued. R1507 (Hoffmann-La Roche) and robatumumab (Merck) also performed poorly in phase II trials (71–73), and several studies were ended early along with the research programs for these drugs.

Some IGF-IR monoclonal antibodies remain in active clinical trials, though. Ganitumab (Amgen), like other drugs of this class, demonstrated promising pre-clinical anti-tumor activity (74, 75), and has moved into phase I and II trials with mixed results. Most of the published phase II trial data have been negative, with no improvement shown with ganitumab treatment for multiple cancer types (76–80). A couple of the phase II trials did produce positive results, though (81, 82), and while no new trials are planned, studies examining this drug’s use in patients with newly diagnosed metastatic Ewing sarcoma, NSCLC, and advanced solid tumors are still actively recruiting. The results from phase II trials for cixutumumab (NCI) have also been mixed, with this agent showing some benefit in patients with thymic epithelial tumors and adipocytic sarcoma, but none in several other cancer types (83–87). Dalotuzumab (Merck) demonstrated anti-tumor activity in pre-clinical and phase I clinical trials of advanced solid tumors (88, 89), but provided no improvement in patient outcome in two phase II studies (90, 91). Both cixutumumab and dalotuzumab remain the subject of a few active phase I and II clinical trials, though, and have been utilized in several completed trials whose results remain unpublished.

#### Receptor-Targeting Agents: Tyrosine Kinase Inhibitors

One hypothesis regarding the IGF-IR antibodies’ poor performance is that IGF-IR inhibition alone allows IR-A signaling to continue and even significantly increase. This may then promote cancer progression, as tumor IR-A expression and activity have been correlated with worse survival (32, 33). Consequently, excess IR-A signaling may be driving resistance to the IGF-IR antibodies. Researchers have posited that one solution to this problem may be dual inhibition of IGF-IR and IR using TKIs. These agents prevent receptor activity by competing for the ATP-binding site in the catalytic domain of the IR and IGF-IR, which is found on the receptors’ beta subunits. Due to the high degree of beta subunit homology between the two receptors, most of these drugs inhibit both IR and IGF-IR activity. Pre-clinical studies demonstrated that one TKI, OSI-906 (Astellas), exhibits anti-proliferative activity on a variety of cancer cell types and enhances the effects of doxorubicin (92, 93). It also performed better than an IGF-IR inhibitor against tumors with both IR and IGF-IR activation (94). The phase I trial results for this agent have been partially positive, with two recent papers reporting observations of anti-tumor activity and good tolerance in patients with advanced solid tumors (95, 96). However, the combination of OSI-906 and the mTOR inhibitor everolimus did not produce an objective response in metastatic colorectal cancer patients (97). In addition, some trials have been terminated early due to safety concerns and/or patient progression, though it appears that some patients are benefiting from treatment with this TKI, as a rollover study is available for subjects from completed trials. There are also active and completed trials that have not yet published their results. BMS-754807 (Bristol-Myers Squibb) and AXL 1717 (Axelar), are two additional TKIs that produced positive pre-clinical data (98–102). Hou et al. (103) demonstrated synergistic inhibition of tumor growth in a mouse model of postmenopausal estrogen receptor positive (ER+) breast cancer following treatment with BMS-754807 plus letrozole or tamoxifen. A phase II trial examining a BMS-754807 plus letrozole combination in aromatase inhibitor (AI)-resistant breast cancer patients has been completed, along with other phase I and II trials, but results have not yet been published. The absence of any new trials with this agent suggests that little to no benefit was produced, though. AXL 1717, which only targets IGF-IR, was tolerated well in a phase I trial for NSCLC. Patients also had indications of a possible tumor response to the drug (104), but published data from other completed trials involving this drug are also lacking. A phase I/II trial for AXL 1717 in patients with recurrent astrocytomas is still recruiting. Overall, as with the IGF-IR antibodies, the TKIs have largely produced disappointing trial results. However, some of the more recent ongoing or completed trials may have benefited from the lessons learned from the early trial failures, including the need for more rational therapy combination. These lessons and future directions based on what we have learned will be discussed in more detail below, following a review of the ligand-targeting agents.

#### Prevention of Ligand Activity

Another approach to the inhibition of IR/IGF-IR signaling is utilization of agents that target the ligands of this system. Insulin secretion and action cannot be directly reduced because this would result in unacceptable metabolic side effects. However, IGF-targeting monoclonal antibodies have been tested pre-clinically and in early clinical trials with some success. Because these drugs cross-react with IGF-I and IGF-II, they result in the inhibition of both IGF-IR and IR-A activity (105–107). Two completed phase I trials for MEDI-573 (MedImmune) demonstrated that this agent is well-tolerated and has some anti-tumor activity in patients with advanced solid tumors (108, 109). Additional phase Ib/II trials for MEDI-573 remain active or are completed but unpublished. Several phase I and II trials testing the safety and efficacy of another IGF antibody, BI 836845 (Boehringer Ingelheim), are currently recruiting patients. While it is too early to declare these drugs more successful than the receptor-targeting agents, their combined inhibition of both IR-A and IGF-IR (but not IR-B) activity suggests that they may lead to an improved clinical response with less metabolic toxicity.

### Lessons learned from clinical trials

#### The Need for Biomarkers

Perhaps the most prominent criticism of past phase II and III trials for insulin/IGF-targeting agents is the researchers’ failure to utilize any predictive biomarkers for the selection of likely responders. To be fair, it should be acknowledged that there were no validated biomarkers for sensitivity to these agents at the start of these trials. In fact, there is still no consensus regarding which biomarker(s) to use for patient selection. However, several have now been proposed and tested in pre-clinical studies. In addition, retrospective analysis of patient biospecimens for the identification of biomarkers linked to drug response has occurred following some trials. These candidate biomarkers can be divided into two categories: insulin/IGF system members and other markers connected to the insulin/IGF axis.

##### Insulin/IGF system biomarkers

One of the first biomarkers proposed was serum IGF-I. Despite the retraction of one study suggesting that elevated pre-treatment free IGF-I levels were associated with NSCLC patient response to figitumumab (110), additional evidence supporting these findings has been published. Figitumumab response in another trial for NSCLC patients was correlated with higher baseline total serum IGF-I concentrations (65). Other IGF-IR monoclonal antibody trials have reported a similar link between circulating IGF levels and drug response (73, 111, 112). In addition, studies utilizing pre-clinical models of multiple forms of cancer have found that response to insulin/IGF-targeting agents is associated with greater IGF-I and IGF-II levels (113–115). While these positive results are encouraging, it is potentially problematic to use systemic IGF levels as biomarkers when tumors are capable of autocrine IGF signaling. Measurement of serum IGFs does not account for local IGF production and could fail to detect tumors that are addicted to autocrine IGF signaling and possibly responsive to insulin/IGF signaling inhibition. While more invasive than a simple blood test, this could be remedied by measuring tumor IGF expression as well as serum levels. Others have also shown that a high IGFBP-5/4 ratio predicted sensitivity to an IR/IGF-IR TKI in a pre-clinical breast cancer model and was correlated with a worse breast cancer outcome, suggesting that this ratio may be indicative of patient response (116). In addition, some studies have reported that tumor IGF-IR expression can predict response (114, 115, 117), but the results have been conflicting (118, 119). Two factors may explain these mixed findings. First, these studies do not account for the possible expression of IR/IGF-IR heteroreceptors, and varying levels of these hybrids versus holoreceptors may also be affecting drug response. Second, there is no standardized protocol for the measurement of tumor IGF-IR expression, so differences in detection methods could contribute to variations in results.

##### Other biomarkers

The remaining candidate biomarkers include gene signatures indicative of elevated tumor insulin/IGF system activity, signaling molecules connected to the insulin/IGF axis that may mediate drug resistance, and other markers signifying drug response (120). In the last category is a pre-clinical study demonstrating that a reduced 2-deoxy-2-[^18^F]fluoro-d-glucose positron emission tomography (^18^FDG-PET) reading following a single dose of the TKI OSI-906 was a marker for cellular sensitivity to this drug (121). This represents a relatively simple method for determining drug response, but may not be practical for trial enrollment since the patient must be started on the drug to measure sensitivity. Others have noted that constitutive Akt and MAPK pathway activation is associated with resistance to the insulin/IGF-targeting drugs (122–124), leading to the idea that the presence of activating mutations in these pathways may be a negative marker for drug sensitivity. However, this idea is confounded by the fact that KRAS mutant NSCLC cells do respond to IGF-IR inhibition (125). This speaks to the complexity of the insulin/IGF signaling system, whose activity can differ by cancer subtype and molecular environment. Finally, an IGF-I gene signature has been identified that was associated with a poor breast cancer prognosis and could be reversed by different anti-IGF-IR agents in the pre-clinical setting. In addition, the gene signature, when combined with measurement of IGF-IR expression, was predictive of IR/IGF-IR TKI sensitivity in breast cancer cells (113, 126). The one clear conclusion that can be drawn from these studies is that more research is needed to test and validate these biomarkers before a consensus can be formed around one candidate.

#### Optimal Drug Combination

The second major criticism of past trials for the insulin/IGF-targeting drugs is that inadequate consideration was given to determining the optimal combination of agents and timing of their administration. Many of the initial trials used the pragmatic approach of simply adding an insulin/IGF-targeting drug to the current standard of care for randomized patient populations where that standard was not performing adequately. This approach, however does not take into consideration the effect previous treatments can have on the biology of the cancer cell, in particular the emergence of resistant cells. Exposure to chemotherapy will kill the majority of cells, but those which can adapt and persist may do so by derailing signaling pathways and reducing their dependence on systems such as insulin/IGF. There is now abundant pre-clinical and clinical evidence that can be used in the design of future trials to achieve improved efficacy. For example, many trials have examined the impact of combining insulin/IGF-targeting drugs to standard chemotherapy regimens, and several phase I trials demonstrated that IGF-IR inhibition is tolerable with standard chemotherapeutic regimens (127, 128). Traditional chemotherapy, in particular platinum-based compounds, targets rapidly dividing cells primarily by inducing DNA damage. Pre-clinical studies suggest IR/IGF-IR signaling protects cells from DNA damage and induces DNA damage repair via non-homologous end joining (NHEJ) and homologous repair (129, 130). Consistent with this, inhibition of IGF-IR causes sensitization to cisplatin (98, 131, 132), doxorubicin, and trabectedin (133), as well as ionizing radiation (129, 134–136) in ovarian, prostate, colon, and breast cancer cells and in mouse xenograft models. Consequently, it may be most beneficial to combine insulin/IGF-targeting drugs with DNA-damaging chemotherapy agents versus those with an alternate cytotoxic mechanism, like the taxanes.

In addition, timing of drug administration may be a key factor in the efficacy of anti-insulin/IGF therapies in combination with chemotherapeutic regimens. Pre-clinical data indicate that the most effective inhibition of tumorigenesis occurs when the drug is administered following chemotherapeutic treatment (68, 92). This is likely due to the fact that IGF-IR inhibition blocks cell cycle progression, thereby reducing the pool of cells being targeted by the chemotherapy. The timing of drug administration was not incorporated into the design of the failed clinical trials. Furthermore, the half-life of the inhibitor should be taken into consideration, as monoclonal antibodies with extended activity may still affect the chemotherapy given in the next treatment cycle. While the clinical trials focused on insulin/IGF system inhibition as a monotherapy or in combination with standard chemotherapy, the effect of inhibition of the system in conjunction with radiotherapy was not addressed. From the abundant pre-clinical data on IGF-IR and DNA damage and repair, such a combination would be appropriate in future clinical trials. As with chemotherapy combinations, certain considerations should be taken in the design of such clinical trials, such as resistance to radiation, timing of therapies, and predictive biomarkers of response. IGF-IR expression may be a useful predictive biomarker for radiation response, as cervical cancer patients with high levels of IGF-IR have a 28.6-fold greater risk of treatment failure (137).

Additional drug combinations have generally been chosen based on IGF-IR’s ability to mediate resistance to inhibitors of other receptor tyrosine kinase (RTK) and hormone signaling pathways. These other pathways can also be upregulated in response to IGF-IR inhibition. The most obvious example of this reciprocal interaction is the increase in IR activity that can occur following IGF-IR inhibition. Reduced IGF-IR signaling results in a pituitary feedback loop that stimulates increased GH secretion, resulting in elevated IGF-I production and hyperinsulinemia (Figure 3). These side effects have been evident in many clinical trials for IGF-IR-targeting agents (89, 138, 139). Insulin, IGF-II, and elevated IGF-I levels can all activate IR-A, driving resistance to IGF-IR antibodies (140–142). Dual IR/IGF-IR TKIs and ligand-targeting antibodies may be able to block this compensatory signaling, leading to improved efficacy over the IGF-IR antibodies (94).

Crosstalk between the insulin/IGF system and ER signaling pathway has been well-established (143–146), and IGF-IR is a known mediator of endocrine therapy resistance in breast cancer (147). The combined inhibition of both pathways has also been shown to be effective pre-clinically (148). Consequently, there was substantial evidence supporting trials examining treatment with IGF-IR antibodies plus endocrine therapy for hormone-resistant breast cancer. The phase II trial results have been disappointing, though, showing no clinical benefit (79). This may be because a complete blockade of insulin/IGF signaling using a TKI or ligand-targeting antibody is needed. Trials investigating the efficacy of an AI plus the TKI BMS-754807 or the IGF antibody MEDI-573 in ER+ breast cancer patients are completed and ongoing, respectively, but no results have been published. Pre-clinical evidence indicates that tamoxifen-resistance is associated with decreased breast cancer cell IGF-IR expression (149, 150), and this would likely reduce the effectiveness of a tamoxifen plus IGF-IR antibody combination treatment. The addition of an insulin/IGF-targeting agent to androgen deprivation therapy for prostate cancer is also supported by pre-clinical evidence, as insulin has been shown to promote androgen production by prostate cancer cells, possibly hastening the development of castration-resistant prostate cancer (151). There are ongoing trials examining the combination of anti-hormone medications with the IGF-IR antibody cixutumumab or the IGF antibody BI 836845.

Finally, several trials have combined insulin/IGF-targeting drugs with EGFR inhibitors in a number of different cancers based on pre-clinical studies showing that EGFR signaling mediates resistance to IGF-IR inhibition and vice versa (152–154). But like so many others, the results from these trials have been largely disappointing, with no clinical benefit shown (7, 73, 77, 90). However, these studies may also have suffered from the failure to utilize biomarkers for patient selection. Some recently completed or ongoing trials for an EGFR antibody plus TKI OSI-906 or IGF antibody BI 836845 combination treatment were open only to NSCLC patients with activating EGFR mutations, which may have improved efficacy. No results have been published yet from the completed studies. Promising data have emerged from *in vitro* and *in vivo* pre-clinical investigations regarding anti-EGFR/IGF-IR inhibitor “nanobullets,” EGFR nanobody liposomes loaded with the IGF-IR inhibitor AG538. This therapy may aid specificity, but would also benefit from stratification of patients through predictive biomarkers (155).

A number of researchers are trying a new direction in their quest to the overcome resistance to the insulin/IGF system inhibitors, looking to the common pathways found downstream of the RTKs for different targets. Specifically, the PI3K/Akt/mTOR pathway has become the focus of intense interest, as a feedback loop allows Akt signaling to continue despite IGF-IR inhibition (156). Conversely, mTOR inhibition also promotes Akt activation through a feedback loop, but this compensatory mechanism can be blocked via IGF-IR inhibition (157). Phase I and II clinical trials combining insulin/IGF-targeting agents with mTOR inhibitors have produced some promising results (118, 158–160) as well as some negative data indicating no clinical benefit (85). Additional phase II trials are ongoing or have been completed, but remain unpublished. One ongoing trial for advanced solid tumors is comparing a ganitumab and everolimus combination with this dual treatment plus the EGFR antibody panitumumab. It appears that many of the current trials are utilizing more carefully considered treatment regimens and screening participants for biomarkers. It remains to be seen whether this translates into better response rates. Interesting pre-clinical data also exist for the combination of insulin/IGF system inhibitors with other drugs, including the lipid modulator simvastatin in prostate cancer (161), the VEGF antibody bevacizumab in ovarian cancer (162), and methyl jasmonate in endometrial cancer (163).

## Energy Balance, Insulin/IGF-I, and Cancer

### Role of insulin and IGF-I in the obesity-cancer link

The prevalence of obesity, defined as having a body mass index [BMI, body weight (in kilograms) divided by height (in meters) squared] ≥30 kg/m^2^, has tripled in the past 50 years in the United States (US). Today, nearly 40% of adults and 20% of American children are considered obese (164). Worldwide, an estimated 750 million people are currently obese (165). Among obese adults, approximately 60% meet the criteria for the metabolic syndrome, a state of metabolic dysregulation characterized by insulin resistance, hyperglycemia, hypertension, and dyslipidemia (166). The hyperinsulinemia induced by insulin resistance is a hallmark of obesity and/or metabolic syndrome (167), and bioavailable IGF-I also increases in the obese state, possibly via hyperglycemia-induced suppression of IGFBP synthesis and/or hyperinsulinemia-induced promotion of hepatic GH receptor expression and IGF-I synthesis (168). Through these mediators, obesity and metabolic syndrome are linked to various chronic diseases, including cardiovascular disease, type II diabetes, and the focus of this review, cancer.

The American Society of Clinical Oncology’s recent position statement on obesity and cancer (169) calls obesity the leading preventable cause of cancer in the US and a central challenge to cancer prevention and care. It estimates that by 2030, 500,000 Americans will be diagnosed with obesity-caused cancers each year unless corrective action is taken. Overall, an estimated 20–25% of all cancer deaths in in the US are attributable to overweight and obesity (170).

### Insulin/IGF-I, inflammation, and cancer

**Figure 4:**
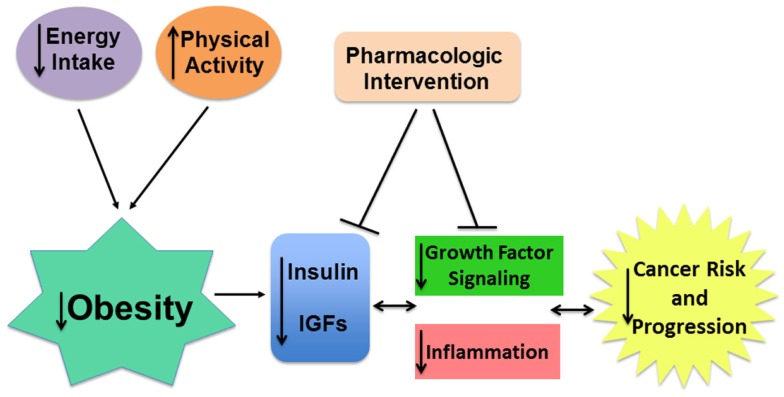
**Targeting insulin/IGF signaling may attenuate the pro-tumor effects of obesity**. Obesity is associated with higher insulin and bioavailable IGF-I levels which, in cooperation with chronic inflammation, contributes to greater risk and progression of many cancers. The pro-tumor effects of obesity can be mitigated directly by obesity reversal using calorie restriction and increased physical activity. Pharmaceutical interventions targeting obesity-associated insulin/IGF and inflammatory signaling or their downstream effectors may also improve patient risk and outcome.

The link between chronic inflammation and cancer development was first noticed more than 100 years ago by Rudolph Virchow, who observed an abundance of leukocytes in neoplastic tissue (171). Now, several tissue-specific inflammatory lesions are established neoplastic precursors for invasive cancer, including gastritis for gastric cancer, inflammatory bowel disease for colon cancer, and pancreatitis for pancreatic cancer (172, 173). In addition to elevated levels of circulating insulin and IGF-I, obesity and metabolic syndrome are associated with a low-grade, chronic (smoldering) state of inflammation characterized by increased circulating free fatty acids and the chemoattraction of immune cells, like macrophages, into the local adipose tissue milieu (174–176). These effects are further amplified by the immune cells’ release of inflammatory cytokines, including interleukin (IL)-1β, IL-6, TNF-α, and monocyte chemoattractant protein (MCP)-1. Adipocytes can enlarge past the point of effective oxygen diffusion, which results in hypoxia and eventually necrosis. Dannenberg and colleagues have established that crown-like structures, rings of activated macrophages surrounding these dead or dying adipocytes, are common in the adipose tissue of obese subjects and are important contributors to the proinflammatory and pro-cancer effects of obesity (177, 178). In addition, free fatty acids escape the engorged/necrotic adipocytes and deposit in other tissues, which in turn promotes insulin resistance and diabetes (through downregulation of IRs and glucose transporters), hypertension, and fatty liver disease. Fatty acid deposition also activates signaling molecules involved in epithelial carcinogenesis, including NF-κB (174–176). This transcription factor is activated in response to bacterial and viral stimuli, growth factors, and inflammatory molecules (e.g., TNF-α, IL-6, and IL-1β) and is responsible for inducing the expression of genes associated with cell proliferation, survival, angiogenesis, metastasis, and further inflammation. Activation of NF-κB is a common characteristic of many tumors and has been associated with insulin resistance and elevated circulating levels of insulin and/or IGF-I (176, 179–181). In summary, there is close reciprocal relationship between obesity-associated elevations in insulin/IGF and inflammatory signaling, such that both factors should be considered in the development of interventions to improve cancer prevention and treatment in the obese patient population (Figure 4).

### Alternative pharmaceutical targets

In addition to pharmacological agents targeting insulin/IGFRs or ligands, including emerging work on microRNA-based approaches (182), a wide variety of natural agents with demonstrated cancer chemopreventive or chemotherapeutic activity have recently been reported to target components of the insulin/IGF pathway (183). These agents, which likely exert only modest inhibitory effects on insulin/IGFR activity, may provide a promising and safe approach, especially if effective combinations can be identified, for breaking the obesity-cancer link.

Pharmacological mTOR inhibitors have emerged as lead candidates for so-called calorie restriction (CR) mimetics, agents that mimic the anti-cancer or anti-aging effects of CR without the restriction of dietary energy intake. Rapamycin treatment extends lifespan and delays cancer in mice, providing additional support for mTOR as a target for mimicking the effects of CR (184). We have shown that rapamycin or its analog, Afinitor^®^ (everolimus), can offset the obesity-associated increased growth of mammary or pancreatic tumors (185–187). Rapamycin and the so-called rapalogs are potent inhibitors of mTOR complex 1, but chronic rapamycin exposure has been linked in some studies to disruption of mTOR complex 2 signaling, resulting in impaired glucose tolerance and insulin action (188). Thus, while inhibiting mTOR complex 1 appears to be a good strategy for mimicking many of the anti-cancer effects of CR, the search for agents that can do so without disrupting mTOR complex 2 signaling is ongoing.

Metformin, a biguanide commonly used to treat type 2 diabetes, is an mTOR-inhibiting drug with great promise as a CR mimetic that overcomes the concerns about glucose intolerance associated with rapamycin/rapalogs. It inhibits gluconeogenesis through indirect activation of AMPK in the liver and may also exert direct effects on AMPK in cancer cells. Administration of metformin suppresses tumor development and/or growth in multiple experimental models, including colon, mammary, and hematopoietic cancer models (189). Epidemiological studies have suggested that type 2 diabetic patients treated with metformin have lower risk of developing or dying from cancer relative to diabetic patients receiving sulfonylureas, insulin, or other therapies (190–192). A randomized trial is now underway to evaluate the effect of metformin on breast cancer recurrence (193). Phenformin, another biguanide that has been abandoned for diabetes therapy due to its toxicity from lactic acidosis, is a more potent AMPK inhibitor than metformin and may also have some potential as a CR mimetic at lower, non-toxic doses (189).

In addition to these pharmaceutical strategies, dietary modulation may also be useful in controlling the high serum IGF-I and insulin levels present in obese cancer patients. While CR is restrictive and difficult to employ, low carbohydrate/high fat diets such as the ketogenic diet can mimic CR in many ways (194) and may be a more amenable dietary alteration for obese individuals at risk for, or diagnosed with, cancer. Low carbohydrate/high fat diets rewire energy metabolism to utilize ketones derived from fatty acids, in particular medium chain triglycerides (MCTs), as an energy source rather than glucose. The ketogenic diet has long been used successfully as a means to reduce epileptic seizures (195) and more recently to regulate blood glucose in type 2 diabetes (196), and studies have shown that the diet has no adverse effects in cancer patients either as an adjuvant or monotherapy (197). Pre-clinical studies using the ketogenic diet have shown promising results in reducing tumor growth in mammary (198), prostate (199, 200), brain (201), and gastric cancer models (202), and it has been shown to promote response to adjuvant therapy (197, 203). In addition to restricting glucose as fuel for tumors, the tumor suppressive effect of the ketogenic diet appears to be mediated through reduction of serum IGF-I and insulin levels via GH resistance (204). Intriguingly, a switch to a low carbohydrate diet can also prevent cachexia in patients undergoing chemotherapy, assisting in the retention of lean body mass (205, 206). The application of low carbohydrate/high fat diets is a promising strategy not only for obesity reversal, but also as a potential treatment in conjunction with normal anti-cancer therapies.

## Conclusion

Insulin and IGF signaling play an important role in the development and progression of many cancers, as they can promote tumor cell proliferation, survival, migration, and invasion as well as angiogenesis. However, despite strong pre-clinical support for their efficacy, most clinical trials testing inhibitors of the insulin/IGF system have produced disappointing results. By increasing the use of validated predictive biomarkers and optimized drug combination treatments, trial outcomes may be improved in the future. Researchers should consider specifically focusing on whether drugs targeting the insulin/IGF system and its downstream signaling molecules may particularly benefit obese cancer patients, who generally have higher circulating insulin and IGF-I levels as well as a greater risk of treatment failure and cancer mortality. There is also a need for more research regarding cancer prevention interventions that counteract the effects of obesity-related elevations in insulin/IGF signaling. Together, these initiatives may lead to a significant reduction in the burden of obesity on cancer risk and mortality.

## Author Contributions

LB, ER, COF, LD, and SH contributed to the writing and revision of this manuscript.

## Conflict of Interest Statement

The authors declare that the research was conducted in the absence of any commercial or financial relationships that could be construed as a potential conflict of interest.
